# Supplementary material for: Are carbon emissions trading and green financial instruments synergistic? -Comprehensive quantitative research based on content analysis
Source: PLoS One. 2024 Mar 7;19(3):e0298601. doi: 10.1371/journal.pone.0298601 (PMC10919601; doi:10.1371/journal.pone.0298601)
Supplement: S1 Data — (ZIP) [file pone.0298601.s001.zip › Data/地级市Z_F工作报告.zip!/╝¬┴╓/═¿╗»╩╨/└┤╫╘╣1⁄2╓┌║┼í╛┬φ┐╦╩2╛▌═°í┐_2017.docx]

各位代表：

　　现在，我代表市政府，向大会作政府工作报告，请予审议，并请市政协各位委员提出意见。

　　一、2016年和本届政府工作回顾

　　2016年，在省委、省政府和市委的坚强领导下，在市人大、市政协的监督支持下，市政府团结带领全市人民，抢抓机遇，主动作为，攻坚克难，奋力拼搏，经济社会发展取得显著成绩，实现了“十三五”良好开局。

　　——经济运行稳定向好。预计地区生产总值实现1040亿元，增长7.5%；地方级财政收入实现82.8亿元，增长6%，增速比上年提高12.7个百分点；社会消费品零售总额实现531.6亿元，增长10%；固定资产投资完成1080亿元，增长10.5%。

　　——动能转换初见成效。大健康产业布局全面展开，医药、食品产业增加值占GDP比重分别达到35%、8.5%，服务业增加值占GDP比重提高1个百分点。新登记企业3301户，增长40.1%。民营经济占经济总量的比重达到53.7%。

　　——对外开放取得突破。抢抓国家“一带一路”倡议机遇，精心谋划向南开放窗口建设，省委、省政府出台了《关于支持通化市建设向南开放窗口的若干意见》。吉林通化国际内陆港务区获批省级经济开发区，创造了当年启动建设、当年通关运营的“通化速度”。

　　——民生福祉持续增进。城镇和农村居民人均可支配收入分别达到23701元、10724元，居民储蓄存款余额增长13.2%。社保、教育、医疗等民生支出200亿元，占公共预算支出79.5%。脱贫攻坚全面推进，35个贫困村、1.6万农村人口实现脱贫。

　　一年来，我们突出稳增长，围绕打好“两个攻坚战”、实现“三个新突破”等重点任务，扎实做了以下工作：

　　（一）着力抓好项目建设和招商引资。组织开展“冬季行动”、春季“三早”行动、“秋季会战”，全市开工5000万元以上项目330个，其中亿元以上项目248个，重大项目数量和质量大幅提高。东宝生物医药产业园、修正医药科技产业园、科泰生物制药产业园等45个10亿元以上项目开工建设，人胰岛素注射剂四期、石油化异地升级改造等续建项目如期竣工。招商引资力度加大，参加“世界500强走进吉林”、“首届全球吉商大会”等招商活动成果丰硕，集中签约招商项目91个、总投资1205.9亿元，其中56个项目落地开工。引进域外资金762亿元，实际利用外资3.96亿美元。

　　（二）着力推动产业转型升级。工业持续稳定增长，规模以上工业实现产值2341亿元、利润151亿元，分别增长9.2%、27%。深入推进工业企业扩能提升，132个技改项目开工，120种新产品实现规模化生产，10户企业签约并购重组，新增规模以上工业企业26户。启动了30个医药健康产业大项目，累计90户医药企业通过新版GMP认证，生物医药特色产业基地通过国家复审，医药产业实现产值1210亿元、增长11.5%。食品、建材、机械制造、化工产业快速发展，冶金、能源产业降幅收窄。服务业攻坚成效明显，鸿丰物流等50个重点项目顺利推进，新增限额和规模以上服务业企业93户，服务业增加值增长10%。太极湾国际旅游度假区、龙溪健康养生谷等26个重点旅游项目加快建设，6个县（市、区）列入国家全域旅游示范区创建名单，旅游收入增长29.1%。1500家电商入驻虎特、苏宁易购等电商平台。

　　（三）着力推进向南开放窗口建设。“三区、两园、两中心、一论坛”平台建设全面启动。国际内陆港务区建设快速推进，通化港公路集装箱中心站、保税仓库、联检大楼134天建成运营，展示了“化不可能为可能”的“通化气魄”。医药高新区发展取得历史性突破，转型升级大幅提速，产业集聚、集群发展格局加快生成，GDP、固定资产投资、地方级财政收入增速明显高于全市平均水平。集安边境经济合作区公路口岸获批国家一类口岸，中韩医药健康产业园、台湾生物科技产业园和东北亚医药健康产品展示中心、跨境电子商务中心建设扎实推进，成功举办第三届长白山国际医药健康产业发展论坛。通集、辉白高速公路开工，通靖高速公路、五月花互通立交建成通车，通化大街、西昌互通立交加快建设。通化至沈阳、至白河、至四平高铁前期工作加快推进。机场扩建列入国家“十三五”机场建设规划。

　　（四）着力深化供给侧结构性改革。“三去一降一补”五大任务有序推进。压缩粗钢产能60万吨，淘汰煤炭产能57万吨。消化存量商品房90万平方米，商品房销售增长256%，列入全省购租并举试点城市。置换政府债务50.5亿元。组建产业基金6支，规模超过200亿元。通化股权托管交易中心获批组建，5户企业在新三板和上海股交中心挂牌。九台农商行通化分行建成开业。列入全省商标质押权登记试点城市。市级国地税合作示范区启动建设，营改增工作全面完成，降低企业成本6.6亿元。申报国家和省科技专项87项，实施中药大品种二次开发项目10项、医药科技攻关和成果转化项目11项。在全省率先向城区下放管理权限87项，向医药高新区授权、委托权限2211项，行政审批实现网上运行。公共资源交易平台和不动产登记中心建成运营。举办了大众创业、万众创新活动周，民营经济主营业务收入增长10%，列入国家东北地区民营经济发展改革示范城市。

　　（五）着力提高城市建设管理水平。实施“城市建设管理提升年”活动，城市功能、市容环境得到明显改善。市区改造棚户区70万平方米，开工率、货币化安置率全省第1，综合整治老旧住宅小区30万平方米；升级改造街路30条，红旗桥拓宽及立交改造主体工程建成通车；官道岭改造全面启动；绿化美化亮化和江南公园、倚江园升级改造、水舞秀音乐喷泉等工程如期完工；建成地下综合管廊10.85公里；列入全省首批海绵城市试点、先行先试房屋租赁试点；施划停车泊位2.6万个，新增新能源公交车50辆，新建公交候车亭34个，智能立体停车场建设有序推进。县（市）公交实现“一卡通”；改造供热管网94公里、供水管网14.2公里、燃气管网14.6公里及2万家庭的老旧燃气设施，天然气母站竣工；建成水冲公厕22座。“五城联创”全面推进，完成121项年度创城任务。“智慧城市”和新型城镇化建设步伐加快。

　　（六）着力加快农业农村和县域发展。粮食增产技术推广面积达到200万亩，粮食产量达到36亿斤。建设标准化牧业小区45个。新增农民专业合作社478家、家庭农场126家。实施千万元以上产业化项目20项，新建特色产业示范基地17个，新创绿色有机无公害产品标识20个，人参、葡萄酒产业产值分别增长30%、17.4%，鲜食玉米、食（药）用菌生产面积分别达到7.5万亩、6000万袋。新增棚膜蔬菜面积4000亩。休闲农业接待人数突破130万人次。农村土地确权和土地收益权贷款试点稳步推进。创建了20个省级美丽乡村、2万个美丽庭院、2万户干净人家。完成水利工程45项。县域经济竞相发展。在全省2013-2015年度县域综合发展考评中，梅河口市获一等奖，通化县、柳河县、集安市获三等奖。我市连续5年被评为全省县域发展组织推动先进单位。

　　（七）着力促进生态文明建设。《通化市生态文明建设示范市创建规划》颁布实施，生态保护红线初步划定，完成污染防治项目19个，淘汰燃煤小锅炉147台，注销黄标车、老旧车4478辆。市区空气环境质量优良天数超过300天。垃圾焚烧发电主体工程完工，城镇污水处理提标改造加快实施。哈泥河水源保护持续推进。植树造林10万亩，清收林地18.4万亩。南山生态移民工程取得新进展。完成了一批地质灾害及矿山地质环境治理工程。整体推进农村环境连片整治经验在全省推广。东昌区、集安市列入国家重点生态功能区，石湖获批国家级自然保护区。

　　（八）着力保障改善民生。“十大民生工程”、100件民生实事如期完成。打响脱贫攻坚战，突出扶贫项目全覆盖，启动“单位包村、干部包户、党员参与帮扶”活动。城镇新增就业4.1万人，农村劳动力转移就业25.3万人。企业退休人员养老金月人均增加132元，城市低保月标准、农村低保年标准分别提高到490元、3500元，城镇居民基本医疗补助标准、新农合筹资标准分别提高到420元、570元。率先在全国地级市全额免除基本殡葬服务费用。市区1.4万户低收入住房困难家庭享受到租赁补贴，分配公共租赁住房1044套。改造农村危房6123户。改扩建幼儿园27所，改造义务教育薄弱校舍5.5万平方米，中小学示范性综合实践基地一期工程和市一中扩建完工。市医院门诊楼开工建设，结核病防治所竣工，建成中医馆42个，我市被确定为全省中医药发展综合改革试验区、医养结合综合试点市、全国第四批城市公立医院综合改革试点市。市科技文化活动中心、全民健身中心、图书馆建成开放，城市规划展览馆完成布展。通化文化云平台成为全国首个城市综合文化云平台。在全省率先实行65周岁以上老年人公交免费乘车。3.1万残疾人享受到生活困难补贴、护理补贴。参加十三届全国冬运会取得3金2银1铜优异成绩。在9天前结束的全国高山滑雪锦标赛上，我市体育健儿勇夺2枚金牌，创1987年以来最佳战绩。安全生产和信访工作进一步加强，事故总量和死亡人数“双下降”，信访积案实现“清零”。市长公开电话受理群众来电6.9万件。食品药品安全形势平稳向好。持续开展严打整治专项行动，平安通化建设扎实推进，人民群众安全感进一步提升。全省公安机关深入推进受案立案和刑事案件“两统一”工作机制改革现场会在我市召开。完善党政军警民合力治边机制，边境安全基础不断夯实。民族团结进步和老龄、妇女儿童等事业全面发展。

　　（九）着力加强民主法治和政风建设。坚持法治通化建设和全国法治城市创建并重，扎实推进依法行政。《通化市城乡规划管理条例》经市人大常委会审议通过、省人大常委会批准施行。办结人大代表建议60件、政协提案136件。深入开展“两学一做”学习教育，凝心聚力、干事创业的正能量进一步彰显。出台了市政府议事规则和抓落实快落实8条规定，行政效率进一步提高。组建软环境建设办公室，专项整治庸懒散等问题，群众、企业的满意度进一步提升。保持了机构编制“零增长”、楼堂馆所“零开工”。查处了一批违法违纪案件，反腐倡廉取得新成效。

　　各位代表！本届政府任期的四年，是通化发展势头好、城乡面貌变化大、人民群众实惠多的四年。四年来，全市上下坚决贯彻省委、省政府和市委决策部署，积极应对经济下行压力加大的挑战，勠力同心，合力攻坚，奋勇前行，付出了艰辛努力，为新一届政府工作打下了坚实的基础。一是综合经济实力明显提升。地区生产总值迈上千亿级台阶，年均增长8.4%；规模以上工业总产值突破两千亿元大关，年均增长14.5%；全口径财政收入、规模以上工业利润均过百亿元，年均分别增长4.1%、16.6%；固定资产投资累计完成3686.9亿元，年均增长14.6%。主要指标增速一直位居全省前列。二是结构调整层次明显提升。三次产业比例由2012 年的9.9：53：37.1调整到8.9：50.6：40.5。医药产业产值年均增长21.2%、四年翻了一番多，总量和效益在全省的占比达到60%。以医药、食品、旅游和山区特色农业为主的大健康产业呈现蓬勃发展态势。三是区位优势和城市功能明显提升。新增通梅、通靖两条高速公路157公里，通丹铁路通车，通化机场通航，海陆空立体交通格局基本形成，实现了由交通末梢到东北东部交通枢纽的重大突破。中心城区规划面积由745平方公里增加到825平方公里，市区城建累计投资220多亿元，完成了供水、供热、供电、燃气管网改造和道路、桥梁等50多项重点工程，370万平方米棚户区经过改造旧貌换新颜，城市承载功能显著增强。四是创新发展能力明显提升。新增规模以上工业企业76户、高新技术企业12户、中国驰名商标8件，上市挂牌企业达到25户。医药高新区晋升为国家级高新区，东宝、修正、紫鑫、万通、四环、科泰、东方等一批大企业、大项目落户医药高新区。五是人民生活水平明显提升。四年完成443件民生实事。城乡居民收入年均分别增长11.4%、10.6%，快于经济增速。教育、卫生、文化、体育等各项事业繁荣进步，城乡养老、城镇医疗、新农合保障实现全覆盖，25万城乡住房困难家庭圆了安居梦。六是对内凝聚力和对外影响力明显提升。获得中国最具幸福感城市、全国首批绿色发展优秀城市、国土资源节约集约模范市、未成年人思想道德建设先进城市、最佳医药投资城市等多项殊荣，创建全国双拥模范城实现“八连冠”。梅河口市列入国家新型城镇化综合试点，集安市、柳河县、通化县、辉南县分别被评为国家园林城市、国家卫生县城、国家生态县、全国休闲农业与乡村旅游示范县，东昌区、二道江区分别被评为全国科技进步先进区、全国法治县区创建先进单位。这些成绩的取得，是省委、省政府和市委正确领导的结果，是市人大、市政协和社会各界鼎力支持的结果，是广大干部群众团结奋斗的结果。在此，我代表市政府，向全市各族人民，向人大代表、政协委员，向各民主党派、工商联、无党派人士和各人民团体，向驻通国省属单位、驻通部队、武警官兵，向所有关心支持通化发展的同志们、朋友们，致以崇高的敬意和衷心的感谢！

　　各位代表！四年的振兴成果鼓舞人心，四年的发展经验弥足珍贵。工作中我们深刻体会到，做好政府工作，必须坚持党的领导，始终把市委决策部署作为总遵循，注重抓落实、求突破，一以贯之、持续发力；必须坚持解放思想，以开阔的视野、开放的思维谋划发展、推动振兴，向改革要活力，向创新要动力，向开放要空间，不断激发振兴发展强大力量；必须坚持发展第一要务，善于抢抓机遇，顶住压力、保持定力，在破解难题中迎难而上，在应对挑战中推动发展；必须坚持以人民为中心的发展思想，以民之所望为施政所向，把保障改善民生作为政府工作的出发点和落脚点；必须坚持“干”字当先、“实”字为要，一张蓝图干到底，一锤接着一锤敲，一步一个脚印地将事业推向前进。

　　在总结工作的同时，我们也清醒地看到，通化发展还存在不少困难和问题。经济总量不大，自主创新能力不强，重点领域改革有待深化，现代服务业发展相对滞后，发展动力转换较慢；创新社会治理面临许多新情况、新问题，安全发展形势不容乐观，维护社会稳定的任务还很繁重；基本公共服务供给依然不足，不能完全满足人民群众不断升级的物质文化需求；市民文明素质和社会文明程度有待进一步提高；政府服务水平仍需提升，一些公职人员主动作为、敢于担当的意识不够强，违法违纪案件仍有发生，发展软环境仍需优化。我们将正视矛盾、直面问题、弥补短板，让全市人民感受到实实在在的变化和成效。

　　二、今后五年奋斗目标和2017年重点工作

　　今后五年，是我市全面建成小康社会的决胜期、绿色转型发展的关键期。国家深入实施东北振兴战略，全省推进长吉图战略向东开放和面向环渤海向南开放双翼共进、打造医药健康支柱产业，为通化发展带来了新机遇新动力新空间。市七次党代会绘就了未来五年宏伟蓝图，为通化率先实现全面振兴指明了方向。奋斗赢得未来，实干才能兴邦。我们要咬定目标，励精图治，奋发图强，干出经得起历史、经得起实践、经得起人民检验的业绩。

　　今后五年政府工作的总体要求是：全面贯彻党的十八大和十八届三中、四中、五中、六中全会精神，认真落实中央“五位一体”总体布局、“四个全面”战略布局、省委“三个五”战略部署和市委“五六七”发展战略，以市七次党代会精神为统领，积极践行五大发展理念，抓牢发展第一要务，深化改革开放，着力改善民生，全面建成小康社会，基本建成吉林省绿色转型发展示范区、东北东部中心城市、国际医药健康名城，努力在东北地区率先实现全面振兴，让全市人民生活得更加美好！

　　今后五年的主要目标任务：

　　——经济实力再上新台阶。发展质量和效益进一步提高，经济增长高于全国、全省平均水平。地区生产总值年均增长7.5%左右，提前两年实现比2010年翻一番。固定资产投资年均增长12%左右，规模以上工业总产值年均增长9%左右，社会消费品零售总额年均增长10%左右，地方级财政收入年均增长7%左右。

　　——发展动能得到新增强。实施支柱产业品质提升、优势产业转型升级、现代服务业提质扩模，构建以大健康产业为引领，医药、食品、旅游三大支柱产业为支撑，山区特色农业为基础，冶金、建材、装备制造、战略性新兴产业加快发展的现代产业新体系，建设创新型城市，打造通化经济升级版。举全市之力推动医药高新区跨越发展，建成全市体制机制创新的示范区、高端医药产业的集聚区、科技创新和转型升级的核心区、经济发展的重要增长极、新一轮振兴发展的排头兵，成为全国医药健康产业发展的一面旗帜。到2021年，大健康产业增加值达到千亿以上规模，服务业增加值占GDP比重力争达到50%，科技进步贡献率提高5个百分点，民营经济占GDP比重达到64%，把通化建成国家产业转型示范区、具有国际影响力的现代医药健康名城、全国重要的绿色食品和功能保健品产业基地、东北亚国际重要旅游目的地、东北东部区域性服务业中心，争创全省山区特色农业转型发展排头兵。

　　——改革开放迈出新步伐。全面深化供给侧结构性改革，经济体制、社会体制、文化体制、生态文明、城市治理、社会治理等重点领域改革取得突破。加快向南开放窗口建设，打造全省国际物流新枢纽、开放发展新引擎、绿色转型发展新高地。推进鸭绿江国际经济合作带和白通丹经济带建设，对接长吉图，密切与环渤海、京津冀和沈阳经济区合作，积极融入中韩自贸区和东北亚经济圈。到2021年，国际内陆港务区吞吐量达到1000万吨。全面提升交通基础设施内联外通水平，实现高速公路“县县通”、中心城市“全辐射”，争取通化至沈阳、白河、四平等高铁开工建设，通化至丹东、集安等高铁项目启动前期工作，推动通化机场扩建为航空口岸机场，加快发展通用航空产业，打造“进关出海、连接腹地、通达国际”的区域性立体交通枢纽。

　　——新型城镇化取得新成效。加强城乡规划、建设、管理，实施“多规合一”，统筹地上地下，实现空间规划与产业布局、城市功能协调统一。壮大通化都市区，彰显品质，打造经典。实施海绵城市、地下综合管廊、生态公园、东南环路等一批提升品位、惠及民生的重点工程，完善市政设施、综合交通网络，完成地下管网升级改造，把通化建成中国北方宜居山水城市。建设智慧城市，建成吉林省东南部云计算中心、东北东部信息交通枢纽。发展特色化、差异化、充满活力的县域经济，打造全省县域经济转型发展示范区。完善县城和重点镇、中心镇基础设施，建设一批美丽宜居乡村，促进城乡一体化发展。

　　——生态质量实现新提升。抓好节能减排、污染防治、生态系统修复、重要生态区域保护，实施大气、水体、土壤清洁行动计划和农村清洁能源工程，推进浑江、鸭绿江、辉发河、哈泥河水系等重点生态廊道综合治理，抓好生态城镇建设，发展绿色产业，构建生态文明制度体系，建成国家园林城市、森林城市、卫生城市、生态文明建设示范市、交通管理模范城市。绿色是万象之源，生态是万业之基。我们要像保护眼睛一样保护生态，像珍惜生命一样珍爱绿色，让通化呈现“高颜值”的蓝天碧水，让通化青山常在、绿水常流、空气常新。

　　——民生社会事业达到新水平。城乡居民收入、各项民生指标走在全省前列。就业、教育、文化、体育、社保、医疗、住房等公共服务体系更加优质化、均等化，在学有所教、劳有所得、病有所医、老有所养、住有所居上取得新进展，人民群众的获得感、幸福感显著增强。打赢脱贫攻坚战，实现“五年任务、两年完成、两年巩固、一年提升”。坚持教育优先，扩大优质学前教育，推进义务教育高水平均衡发展，普及高中阶段教育，加快发展现代职业教育。支持通化师范学院建设应用型本科高校。深化医药卫生体制改革，推进医疗、医保、医药联动，完善多层次医疗卫生服务体系和现代医院管理制度，率先迈入国家级健康城。加快发展养老养生产业，建设国际养生休闲度假基地。提高城市文化软实力，彰显民俗文化、红色文化、生态文化、产业文化等历史文化魅力，扎实开展全国文明城市创建。

　　——社会治理呈现新局面。加强基层社会治理，完善重大决策社会稳定风险评估机制，构建全民共建共享的社会治理格局。健全民主法制，保障公共安全。建设平安通化、法治通化，创建全国法治城市。争创全国双拥模范城“九连冠”。

　　“为者常成，行者常至”。未来五年，我们一定盯紧目标，善做善成。勤劳智慧的通化人民，一定能用激情和汗水，谱写率先实现全面振兴的辉煌篇章！

　　各位代表！新开局要有新气象，新起步要有新作为。今年是新一届政府履职的第一年，我们要认真贯彻中央和全省经济工作会议精神，全面落实市七次党代会部署要求，坚持稳中求进工作总基调，适应把握引领新常态，全面做好稳增长、促改革、调结构、惠民生、防风险各项工作，促进经济平稳健康发展、社会和谐稳定。预期地区生产总值增长7.5%左右，地方级财政收入增长7.5%左右，固定资产投资增长12%左右，社会消费品零售总额增长10%左右，居民消费价格涨幅3%左右，城镇登记失业率控制在4.5%以内，城乡居民人均可支配收入稳定增长，节能减排完成省下达任务。重点做好九个方面工作：

　　（一）持续扩大有效投资。实施5000万元以上项目330个、亿元以上项目240个。开工建设东方生物制药、绿邦医药生产及医疗数据等新项目，加快建设修正医药科技产业园、紫鑫医药产业园、科泰生物制药及功能食品等续建项目，推动东宝重组人血白蛋白注射剂、万通40亿片感通片等项目建成投产。主动对接东北振兴若干意见、“国14条”等相关政策，包装储备亿元以上项目130个，争取更多项目列入国家专项。创新投融资体制，鼓励民间投资，提高民间投资规模，扩大PPP模式运用。精心谋划招商项目，有效实施精准招商，促进签约项目落地，抓好十一届东博会等重大活动招商引资，引进域外资金增长10%，实际利用外资增长8%。5000万元以上项目实行清单式管理，强化服务调度，推动项目落地开工、达产达效。继续开展“冬季行动”、春季“三早”行动、“秋季会战”、项目集中巡检，新建续建项目上半年开工率分别达到80%以上、100%。

　　（二）加快产业结构调整步伐。推动实体经济转型升级，滚动实施工业扩能提升工程，推进15户企业并购重组，抓好156个重点技改项目、120种新产品规模化生产。推动“银政企保”对接，加强产融合作，搞好要素保障、运行组织，扩大药品、钢材、建材等地产品互采互用，确保工业稳定增长。规模以上工业总产值增长9%左右。培育规模以上工业企业20户。推进大健康产业5年行动计划，谋划实施总投资超过2000亿元的产业集群项目。推动新药进医保目录，搞好大品种技术升级与二次开发，做强现代中药，做优生物制药，做大化学制药，加快构建医药工业、医药商贸、医药科研、医药教育、中药材基地、医药康复、医药文化“七位一体”现代医药产业体系。深度开发长白山特色食品资源，抓好吉林农牧食品、康元林蛙、禾韵蓝莓等30个超亿元项目，提升绿色食品、功能保健品品质。启动葡萄酒产业振兴计划，擦亮通化葡萄酒品牌，重点打造鸭绿江河谷和柳河山葡萄酒产区，葡萄酒产业产值增长10%以上。支持通钢去产能、去杠杆，争取债转股试点。推动二道江区产城融合发展，推进江东老工业区搬迁改造，推广扩大玄武岩、石墨等高性能复合材料应用，加快建设石油装备、汽车配件、化工等特色园区。申报中国制造2025试点城市。开展消费品工业“三品”专项行动，增加中高端产品供给。建设四环食品药品检验检测中心，筹建国家级人参检验室，提升国家果酒及果蔬饮品质量监督检验中心功能，做好国家食品药品安全示范市创建工作。新创一批驰名商标、名牌产品、地理标志保护产品。促进医药、食品、钢材等工业制成品出口，实现外贸进出口总额3.4亿美元。

　　继续打好服务业发展攻坚战。启动实施“三个一百”工程，新增限额和规模以上服务业企业100户，抓好100个服务业重点项目，包保100户服务业重点企业。服务业增加值增长10%，占GDP比重提高1个百分点以上。着力发展旅游业，加快鸭绿江国际旅游带、大长白山旅游圈综合开发，实施通化滨水欢乐谷、龙堡四季旅游度假区等24个重点旅游项目，力争高句丽文物景区晋升5A级景区，扎实创建国家全域旅游示范区、中医药健康旅游示范区、边境旅游试验区；实施冰雪旅游产业升级工程，推进万峰滑雪旅游度假区、果松国际滑雪场等项目早日开工建设；发展红色旅游，完善抗联精品线路，新建集安抗美援朝第一渡纪念公园等红色景区，保护、修复红色历史遗迹；筹建旅游集散中心，发展智慧旅游，办好旅游节庆活动，加强宣传促销，推动文化旅游、乡村旅游、森林旅游、健康养生旅游协调发展，旅游收入增长30%以上。集聚发展现代物流业，推动建设保民祥医药物流中心、鸿丰物流园区二期工程，做好国家市场采购贸易试点申报。发展“互联网+” 新产业、新业态，抓好东北亚跨境电子商务平台、“通药网”医药电子平台、县域电子商务服务中心、“淘宝村”建设，电子商务网络零售交易额增长50%以上。

　　（三）扎实做好农业农村工作。深入推进农业供给侧结构性改革，优化农业产业体系、生产体系、经营体系。调减籽粒玉米种植5万亩，发展鲜食玉米7.5万亩，推广粮食增产技术面积100万亩。完成基本农田划定工作，建设高标准基本农田3.9万亩。加快发展现代畜牧业，建成标准化牧业小区40个，免疫无口蹄疫区建设通过国家评估验收。实施农特产业基地建设工程，规划建设东昌和二道江环城农业产业带、集安和通化县特色农业示范区、梅柳辉农牧结合区。抓好康美、卓越等20个产业化重点项目，农产品加工业实现产值590亿元，推动一、二、三产业深度融合。加快发展中药材、食（药）用菌、红松果林、山核桃等特色产业。实施人参产业振兴工程，推进人参种植规范化、质量标准化、产业集群化、品牌国际化、市场全球化，建设一批超亿元人参产业项目，提升人参电子交易平台功能，人参产业产值增长20%。新建棚膜蔬菜4000亩。新创20个绿色有机无公害产品标识，打造通化大米、人参、山珍等知名品牌。加强农产品质量监管。培育壮大专业大户、家庭农场、农民合作社、休闲农庄等新型经营主体，农业适度规模经营比重达到45%以上。实施专门行动计划，促进农民稳定增收。抓好江河堤防整治等49项水利工程。规划建设一批特色宜居小镇。启动改善农村人居环境四年行动计划，建成10个省级美丽乡村，创建1.4万个美丽庭院、8000户干净人家。

　　（四）加速打造吉林省向南开放新窗口。加快通集、辉白高速公路建设，推动通化至沈阳、至白河、至四平高铁尽早开工，做好通化至丹东、集安高铁和机场扩建前期工作，争取实现境外包机运行。西昌互通立交建成通车。强化与丹东港深度融合，完成通化港铁路集装箱中心站建设，实施二密站扩能改造，力争建成保税物流中心（B型），国际内陆港务区吞吐量达到500万吨。加快建设中韩医药健康产业园、台湾生物科技产业园、东北亚医药健康产品展示中心，推动各类开发区竞相发展。医药高新区地区生产总值增长15%，引进、建设亿元以上项目超过60个。将长白山国际医药健康产业发展论坛纳入东博会。做好集安国家级边境经济合作区申报，实现中朝界河大桥和公路口岸联运通行。推进白通丹经济带发展规划实施，促进东北东部区域合作。搞好与浙江的对口合作。

　　（五）切实提升城市品位和承载功能。坚持人民城市人民建、人民城市人民管、人民城市为人民，以更大的力度推动城市建设、强化城市管理。编制完善新一轮城市总体规划、土地利用总体规划、综合交通规划、主体功能区规划、中心城区控制性详细规划、历史文化遗存保护规划，突出“多规合一”，强化规划法定作用。市区改造棚户区65万平方米，综合整治老旧住宅小区30万平方米。开工建设新城路、玉泉路等5段地下综合管廊，形成廊体6.56公里。推进海绵城市建设，争取国家试点。升级改造建设大街等25条街路巷路，加快官道岭综合改造，完成通化大街9.6公里改造、红旗桥立交收尾工程。新建2座智能立体停车场、10座水冲公厕，新增30台新能源公交车。实施调峰热源环保扩容，新建15座换热站，改造燃气、供水管网17公里，新增自来水和燃气用户8000户，改造室内老旧燃气设施6万户。加快筹建城市备用水源、第二净水厂。改造三期平湖工程，提升城区水域景观。抓好绿化美化亮化。续建新建龙岗山、江南湿地等10处公园。深化经营城市理念，推进棚改地块土地收储，力求土地增值最大化。加强城市管理，在精细、标准、规范上下功夫。抓好环境卫生、户外广告、指示牌、城乡结合部、城区养犬等专项整治，强化住宅小区违建整治、重点区域违建管控。推动物业管理重心下移，物业管理覆盖率达到80%。加快建设智慧城市，抓好大数据中心、公共基础数据库、智慧管网子系统等项目。强力推动“五城联创”，国家园林城市、森林城市通过检查验收，力争实现全国交通管理模范城市创建。

　　（六）大力推进改革创新。以供给侧结构性改革为主线，突出抓好基础性、关键性改革。深入推进“三去一降一补”，压减通钢炼铁产能80万吨，15万吨以下煤矿全部退出。加大棚改货币化安置力度，提前完成商品住房去库存三年任务，引导房地产市场健康发展。创新金融服务，优化信贷投放，提高直接融资比重，推动政府存量债务置换，筹建金融街区，实现股权托管交易中心挂牌运营，积极推进企业上市，完成农信社改制，力争组建3家村镇银行，争取设立民营银行，引进域外银行、保险、投资机构等金融资源，守住不发生区域性、系统性金融风险的底线，让金融更好浇灌实体经济。深入开展清费减负，进一步减轻企业负担。加快市级国地税合作示范区建设。统筹抓好国有企业家属区“三供一业”分离移交工作。稳步实施厂办大集体改革。纵深推动“放管服”改革，开展优化投资营商环境、减证便民等专项行动，把该“放”的彻底放开、该“减”的彻底减掉、该“清”的彻底清除。加强行政权力运行和事中事后监管，动态调整权力清单、责任清单，建成全市政务服务“一张网”。完善“双随机、一公开”监管模式，做到执法领域全覆盖。实施城市执法体制改革，深化“多证合一”等商事制度改革，稳步推进供销合作社综合改革，全面完成土地确权登记和国有林场改革任务，统筹推进财税、事业单位、户籍、公车等改革。推动社会信用体系建设，打造诚信通化。开展中小企业入规升级专项行动，实施企业家素质提升工程。民营经济主营业务收入增长10%。

　　深入实施创新驱动战略。争取列入国家、省科技支持项目50项，深化与“十四校三所一院”合作对接，组建医药健康、葡萄酒、保健品等产业创新联盟，完善新药研发、技术转化中试平台，推动重大科技成果在通化转化。实施科技小巨人企业育成计划，加快建设双创中心等众创空间、孵化园区，促进大众创业、万众创新。加强人才队伍建设，举办职业技能大赛，建立技能大师工作室，培养高素质技能型人才，积极引进高层次人才。

　　（七）加强生态环境保护建设。实施蓝天工程。落实大气污染防治计划，抓好冶金、火电等行业超低排放改造，整治燃煤小锅炉、机动车尾气和扬尘污染，推进柳河、通化县秸秆综合利用，淘汰黄标车。城市空气环境质量优良天数比例达到80%以上。实施碧水工程。加强水资源管理，推行河长制。推进玉带河、柳条沟黑臭水体治理，完成10个重点流域污染防治项目。提标扩建污水处理厂，加快哈泥河饮用水源和森林、湿地保护区生态环境综合治理。建设鸭绿江、浑江等生态水网，实施浑江城区段清淤工程，优化提升滨江亲水环境。治理水土流失面积160平方公里。启动周山侵蚀沟综合整治。重点流域水质优良率达到70%以上，饮用水源水质优良率达到100%。实施青山工程。造林8万亩，省级标准化绿化村屯4个，确保无重大森林火灾。实施净土工程。开展污染源普查，抓好土壤污染防治，推动农药化肥施用量零增长。推进矿区和地质环境综合整治。强化环境执法，健全完善考评追责机制，实现环境监管全覆盖。

　　（八）协调发展各项社会事业。改造中小学薄弱校舍4万平方米。在全省率先通过国家义务教育均衡发展整体评估。加强校地合作，筹建医药健康职业学院。稳步推进城市公立医院综合改革，加快建立分级诊疗制度，市中心医院创建“三甲”医院，提高基层卫生服务能力，确保医改“惠民强医”。建成市医院门诊楼。在全省率先推广使用居民健康卡，争创国家医养结合试点市、基层中医药工作先进市。稳妥实施全面两孩政策。推进文化惠民工程，加快公共文化服务体系建设，组织送戏下乡300场，举办广场演出50场。筹排实景演出《印象通化》，创排话剧《回家、宝贝》。强化文化遗产保护传承，推进自安山城、万发遗址公园建设，承办中国文化遗产日吉林省主场城市活动。培育发展新兴文化产业，办好松花砚（石）文化节。备战十四届全国冬运会、十八届省运会，建成滑冰馆。举办全市田径运动会。社区、行政村健身器材力争实现全覆盖。推动民族团结进步。做好宗教工作。保障妇女儿童权益。发展老龄、残疾人事业。支持驻通部队建设，加快军民融合深度发展。

　　（九）持之以恒改善民生。为政之道，民生为本。我们要念之再三、铭之肺腑，多谋民生之利，多解民生之忧。集中力量办好民生实事。坚持精准扶贫、精准脱贫，确保38个贫困村、1.2万人实现脱贫。落实就业促进计划、创业引领计划，城镇新增就业3.9万人，农村劳动力转移就业22万人。开展全民参保登记计划，按政策提高基本养老金、失业保险金、城镇医保、新农合补助标准、低保对象大病救助标准，城市低保标准不低于上年度城镇居民人均可支配收入的20%，农村低保标准不低于脱贫计划确定的最低指导标准，确保养老金正常发放。整合城镇居民医保和新农合，扩大异地就医直接结算。深入开展“情暖万家”、“圆梦大学”等慈善救助活动，使困难群众遇急有助、遇困有帮，让通化这个大家庭充满关爱和温暖。规范完善社区居家养老服务，筹划社会福利院异地新建，抓好优抚医院、老年医院、医疗养老关爱护理中心等项目，争创居家养老国家试点城市。优化社区管理资源，强化社区服务功能。加快“城中村”改造。为市区1.3万户低收入住房困难家庭发放租赁补贴，改造农村危房4859户，让住房困难群众实现“居者有其屋”。抓好农村饮水安全巩固提升。

　　促进社会平安和谐。牢固树立红线意识，统筹推进安全生产领域改革，深入开展“安全生产责任深化年”活动，启动企业主体责任落实“五个一”工程，突出重点领域安全防控和整治，坚决遏制重特大事故发生。深入实施食品药品放心工程，保障人民群众“舌尖上的安全”。实行联合接访、局长接待日制度，依法推动信访问题及时就地解决，保持信访积案“清零”态势。完善立体化、信息化社会治安防控体系，严厉打击暴恐犯罪、经济犯罪、网络电信诈骗等活动，提高人民群众安全感。抓好“七五”普法，推进依法治市。加强应急管理。做好防汛抗旱、地震防灾减灾等工作。强化边境联管联控，确保边境安全稳定。

　　三、加强政府自身建设

　　重任千钧惟担当。面对繁重的改革发展任务，我们一定要加强和改进政风，把振兴发展的责任和使命牢牢扛在肩上，把万家忧乐放在心头，努力建设人民满意的政府。

　　坚持为民执政。“天地之大，黎元为先”。各级政府公职人员要牢记全心全意为人民服务的根本宗旨，始终把人民利益放在心中最高位置，时刻与群众保持血肉联系，尽心竭力为群众办实事、解难题、谋福祉，让全市人民的生活年年都有新变化、一年更比一年好。

　　着力依法行政。把政府工作全面纳入法治轨道，尊法、学法、守法、用法，依法履职，依法办事，建设法治政府。全面提高法治思维、法治方式，重法治、严规范，法定职责必须为，法无授权不可为。自觉接受人大法律监督、工作监督和政协民主监督，主动接受社会监督、舆论监督，让权力在阳光下运行。

　　着力提升效能。坚持打基础、利长远，办实事、求实效，恪尽职守、夙夜在公，雷厉风行、锲而不舍。构建“亲”、“清”政商关系，尊重企业家，善待企业家，包容企业家，营造“帮忙不添乱、办事不求人”的发展环境，让群众和企业少跑腿、好办事、不添堵，为人民群众和广大投资者创造服务最优、效率最高的软环境。

　　着力推动落实。建立工作清单，全程督查督办，完善考评机制，强化绩效评估，严格追责问责，以抓铁有痕、踏石留印的劲头，一抓到底。坚决整肃懒政、怠政，坚决惩处失职、渎职。减少会议和文件，办有效的事，开务实的会，发管用的文。深入调查研究，确保工作接地气。健全容错纠错、正向激励机制，给改革创新者撑腰鼓劲，让全体公职人员想干事、会干事、干成事。

　　着力从严治政。加强作风和能力建设，深入践行“三严三实”，严格执行中央八项规定和省委、市委具体规定，驰而不息纠正“四风”。严肃党内政治生活，强化党内监督，立身不忘做人之本，为政不移公仆之心，用权不谋一己之私。心存敬畏，手握戒尺，以上率下，建设廉洁政府。

　　各位代表！蓝图已绘就，扬帆正当时。让我们在市委的坚强领导下，在市人大、市政协的监督支持下，以更大的担当、更实的作风、更足的干劲，不忘初心，团结拼搏，开拓进取，为率先实现全面振兴、让全市人民生活得更加美好而努力奋斗！
